# Microglia in Alzheimer's Disease

**DOI:** 10.1155/2012/598371

**Published:** 2012-11-28

**Authors:** Lee-Way Jin

**Affiliations:** Department of Pathology and Laboratory Medicine, Alzheimer's Disease Center and UC Davis MIND Institute, Davis, 2805 50th Street, Sacramento, CA 95618, USA

This special issue describes exciting papers contributed to the International Journal of Alzheimer's Disease issue on microglia in Alzheimer's disease (AD). It has been well recognized that neuroinflammation is an early event in the pathogenesis of AD, in which microglia is a major player. A core theme of the articles presented in this special issue is the dichotomous role played by the microglia—the context-dependent neuroprotective and neurotoxic functions. Recent insights regarding molecular mechanisms that regulate the diverse microglia responses are summarized. The understanding of these molecular pathways would lead to targeted therapies aimed at modulating microlgial behavior toward neuroprotection, as exemplified by approaches targeting the microglial KCa3.1 potassium channel, scavenger receptors, or complements, for example. Microglia also play critical roles in comorbidities of AD, such as diabetes and traumatic brain injury.

The first paper I would suggest the readers to read is R. E. Mrak's neuropathological survey of microglia in Alzheimer brain. Thanks to the pioneer observations by neuropathologists about two decades ago, microglia emerged into the scene of AD research. Although our view of microglia in AD has since substantially changed, mostly based on studies using rodents as models, human neuropathology still provides a “golden standard” or reality check for what is relevant and important to AD. Interesting sets of data presented by R. E. Mrak suggest that microglia actively influence the pattern of development of amyloid plaques as well as neuronal tangle formation in AD brains, rather than simple associations with these lesions. In addition, plaques of different stages, perhaps reflecting different pathological stages of AD, are associated with activated microglia with different morphology or immunohistochemical phenotypes. The readers can consult the article by D. M. Wilcock for a synopsis of various states of microglia that may serve different functions. In principle, microglia possess all the response repertoire of peripheral macrophages; therefore the established classification system for the inflammatory state of macrophages, based on defined sets of molecular markers, can be applied to microglia. D. M. Wilcock further provides a summary about how the microglia inflammatory state can be altered by specific sets of stimuli, how different states influence amyloid load, and how passive immunization with anti-A*β* antibodies alters the microglia inflammatory state prior to significant reductions in amyloid deposition. Again, this line of evidence supports an active role of microglia in the development and dissolution of amyloid plaques. However, how the microglia inflammatory phenotypes related exactly to the pathologies of AD appears to be complex and remains to be explored.

In this issue, M. Noda and A. Suzumura provide a comprehensive review of the molecular players in the quintessential functions of microglia—chemotaxis and phagocytosis. They point out that the lack of microglial phagocytosis worsens the pathology of AD and induces memory impairment. In addition to the well-known players such as phosphatidylserine receptors, scavenger receptors, and complement factors, M. Noda and A. Suzumnura also discuss a less publicized but highly interesting phagocytosis modulator expressed in microglia—*γ*-secretase, which in neurons is certainly the center of attention for its role in generating A*β*. One novel phagocytosis modulator relevant to AD reported in this special issue is the high mobility group box protein 1 (HMGB1). HMGB1 is a nonhistone chromosomal protein that is released from cells undergoing necrosis and acts as an inflammatory mediator. K. Takata et al. report evidence to support that microglial A*β* phagocytosis dysfunction may be caused by HMGB1 that accumulates extracellularly on A**β** plaques.

When it comes to the effect of microglial phenotypes on neurons, microglia serve a biphasic role in AD—they are either neurotoxic or neuroprotective. In this issue, T. Mizuno reviews the molecules that trigger and execute the neurotoxic actions of microglia in AD, as well as those able to induce microglia neuroprotective properties. However, the interwoven molecular pathways and the dichotomous behavioral pattern of microglia make it difficult to provide a clear answer regarding if a microglia action is beneficial or harmful. A synthesis of the complex clinical and experimental literature encompassing the debate of “good” and “bad” microglia (and, of course, without excluding the possibility of microglia playing no role in AD) is provided by T. M. Weitz and T. Town. They conclude that an understanding seems possible when considering context—the conditions under which microglia encounter AD-like pathological lesions. Specifically, a model emerges where microglia mount different types of activated responses depending on whether they encounter particular species of misfolded protein (A*β* or tau) and whether this innate recognition occurs early on or after pathology is well established, an insight that echoes the neuropathological observations reviewed by R. E. Mrak.

A group of well-studied and highly significant molecules in microglia is scavenger receptors (SRs), reviewed by K. Wilkinson and J. El Khoury. Although the roles of several members of SRs in AD are diverse and complex, convincing evidence shows that SCARA-1-mediated microglia interactions with A*β* are beneficial and promote phagocytosis and clearance of A*β*, whereas CD36-A*β* interactions are harmful and together with TLR-4 and TLR-6 lead to the production of neurotoxins and proinflammatory molecules. The other group of molecules reviewed in this issue is complements. H. Crehan et al. review evidence indicating that the ability of microglia to adopt the “good” or “bad” role in AD is influenced by complement factors. Furthermore, microglia appear to be a major player in the dysregulated complement cascade in AD, in which microglia are aberrantly activated by disruptive complement signaling and A*β* to enhance their secretion of cytokines, which can lead to further complement cascades.

Along this line of research on “good” and “bad” microglia, I. Maezawa et al. actually propose a molecular target that is ready for manipulation by pharmacologically well-characterized small molecules. They provide evidence that targeting the microglia potassium channel KCa3.1 could harness calcium signaling resulting from microglia activation, thus “fine tune” the microglia response toward neuroprotection—phagocytosis of A*β* with reduced release of harmful inflammatory mediators and oxidative species. Because the pharmacology of KCa3.1 has been well studied and highly selective inhibitors are available and safe, targeting KCa3.1 may hold promise as a specific anti-inflammatory therapy for AD.

Also included in this special issue are two articles discussing the microglia link in two significant comorbidities in AD: traumatic brain injury (TBI) and type II diabetes mellitus (T2DM). R. C. Mannix and M. J. Whalen point out that A*β*, which is elevated acutely after TBI, may be a key mediator of microglial activation in TBI. TBI-induced A**β** alters long-term microglial function and A**β**-clearance and could therefore contribute to the development of AD. In this regard, the advancement of drug discoveries in the field of TBI, such as sex steroids or Apoe mimetics which alter both microglial function and A**β** metabolism, may have potentially important roles in TBI as well as AD. Microglia-mediated neuroinflammation appears also to provide a shared mechanism between AD and T2DM. L. F. Lue et al. review evidence supporting that T2DM could accentuate microglial activation, neuroinflammation, and vascular inflammatory/oxidative injury in AD brains through mechanisms mediated by RAGE and other pattern recognition receptors and the cascade of cytokine and chemokines. Because a wide range of inflammatory mediators and receptors are involved in these two diseases, it is important to characterize the patterns of microglial activation in AD patients with T2DM and AD patients without T2DM for future anti-inflammatory approaches. One interesting difference pointed out by L. F. Lue et al. is that complement activation is a prominent feature in AD, but not in T2DM.

In summary, common themes emerge in the articles presented in this special issue. Microglia response is highly complex in AD and is context dependent. Aging, the most significant risk factor for AD, and other risk factors such as TBI and T2DM may contribute to the development of AD via altered microglia-mediated neuroinflammation (microglia dysfunction). The task ahead is to be able to manipulate microglia responses to therapeutic advantage. Further understanding of the triggers and molecular pathways of various microglia responses will help us to design microglia-targeted therapies to enhance the neuroprotective or the “good” behavior and at the same time dampen the neurotoxic or the “bad” behavior of microglia.

*Lee-Way Jin* *Lee-Way Jin*

